# Spatial and temporal variations of air quality and six air pollutants in China during 2015–2017

**DOI:** 10.1038/s41598-019-50655-6

**Published:** 2019-10-23

**Authors:** Hong Guo, Xingfa Gu, Guoxia Ma, Shuaiyi Shi, Wannan Wang, Xin Zuo, Xiaochuan Zhang

**Affiliations:** 1 State Key Laboratory of Remote Sensing Science, Institute of Remote Sensing and Digital Earth, Chinese Academy of Sciences, Beijing, China; 2 University of Chinese Academy of Sciences, Beijing, China; 3 Chinese Academy for Environmental Planning, Beijing, China

**Keywords:** Environmental impact, Environmental economics

## Abstract

Air pollution has aroused significant public concern in China, therefore, long-term air-quality data with high temporal and spatial resolution are needed to understand the variations of air pollution in China. However, the yearly variations with high spatial resolution of air quality and six air pollutants are still unknown for China until now. Therefore, in this paper, we analyze the spatial and temporal variations of air quality and six air pollutants in 366 cities across mainland China during 2015–2017 for the first time to the best of our knowledge. The results indicate that the annual mean mass concentrations of PM2.5, PM10, SO_2_, and CO all decreased year by year during 2015–2017. However, the annual mean NO_2_ concentrations were almost unchanged, while the annual mean O_3_ concentrations increased year by year. Anthropogenic factors were mainly responsible for the variations of air quality. Further analysis suggested that PM2.5 and PM10 were the main factors influencing air quality, while NO_2_ played an important role in the formation of PM2.5 and O_3_. These findings can provide a theoretical basis for the formulation of future air-pollution control policy in China.

## Introduction

Air pollution has become a serious global threat to human health and welfare^1,2^. With the rapid development of China’s economy, more fossil fuels are burned and vehicles are in use, resulting in increased particulate matter (PM2.5 and PM10), carbon dioxide (CO_2_), nitrogen dioxide (NO_2_), sulfur dioxide (SO_2_), and ozone (O_3_) concentrations in the atmosphere^3^. Air pollution has already aroused significant public concern in China, especially in recent years^4–6^. Air-pollution data with high spatial and temporal resolution are needed to accurately evaluate the health risks associated with air-pollutant exposure^7^. Fortunately, hourly concentrations of PM2.5, PM10, SO_2_, NO_2_, CO, and O_3_ have been published online by the Ministry of Environmental Protection (MEP) since January 2013.

Spatial and temporal variability of air pollutants are key parameters in the assessment of associations between exposure and human health^8^. A number of studies have reported temporal and spatial variations of gaseous and particulate matter pollutants in China^9–11^, but most of them were limited only to a single city or air pollutant^12^. For example, Zhang and Cao^13^ revealed the spatial and temporal variability of PM2.5 concentrations in 190 cities from April 2014 to April 2015. Cheng *et al*.^14^ indicated that the annual mean NO_2_ concentrations decreased significantly from 71.0 µg/m^3^ in 2000 to 49.0 µg/m^3^ in 2008.

Spatial and temporal variations of multiple pollutants have also been reported^15,16^. Wang *et al*.^7^ indicated that PM2.5, PM10, CO, and SO_2_ concentrations were higher in cities located in the North region than those in the West and Southeast regions in 31 capital cities from March 2013 to February 2014. Yan *et al*.^17^ pointed out that PM2.5 was the most serious pollutant, followed by O_3_ in Beijing in 2013. Although the previous studies provided valuable insights, none covered long-time air quality and data of the six air pollutants to reveal the yearly variations, and the cities in the aforementioned studies were too few to represent the characteristic of air quality and the six air pollutants in China.

The monitoring sites of air pollutants have been stable in 366 cities in China since January 2015. Therefore, the purpose of this study is to understand the temporal and spatial variations of PM2.5, PM10, SO_2_, NO_2_, CO, and O_3_ in 366 cities from 2015 to 2017. We analyzed the variations of air quality index (AQI) and six air pollutants during 2005–2017, and revealed the correlations among air pollutants under different AQI ranges using Pearson’s correlation coefficient.

## Results

### Overview of AQI and six air pollutants

**Figure 1:**
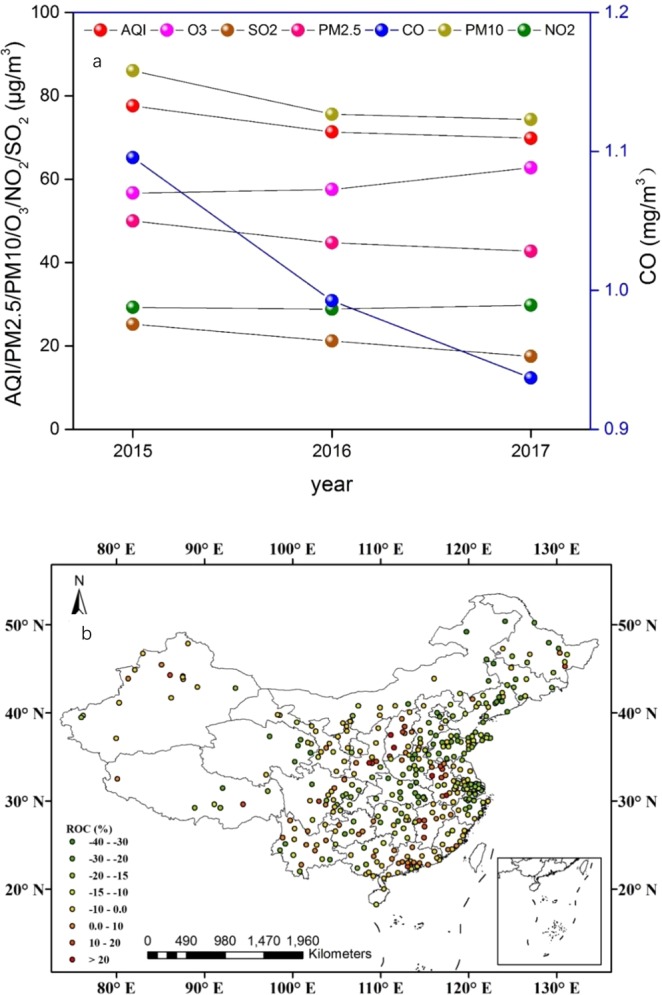
(**a**) Overview of variations of AQI and six pollutants over mainland China during 2015–2017; (**b**) Regional differences in annual mean AQI over mainland China between 2017 and 2015.

The annual mean AQI has decreased year by year over mainland China (Fig. 1a). Compared with 2015, it decreased by 10.0% in 2017. Further analysis indicated that the air quality improved in 298 cities, and the number of cities with excellent air quality (see Supplementary Material, Table S1) increased from 46 in 2015 to 64 in 2017, while that with slight pollution decreased from 49 in 2015 to 26 in 2017 (Fig. 1b). The annual mean mass concentrations of PM2.5, PM10, SO_2_, and CO all decreased year by year. Compared with 2015, they decreased by 14.5%, 13.6%, 30.5%, and 14.5% in 2017, respectively. Different from the four air pollutants, the annual mean NO_2_ concentrations were almost unchanged. However, the annual mean O_3_ concentrations increased year by year, e.g., by 10.7% in 2017 compared with that in 2015.

### Spatial and temporal variations of six air pollutants

#### Variations of PM2.5 concentration

**Figure 2:**
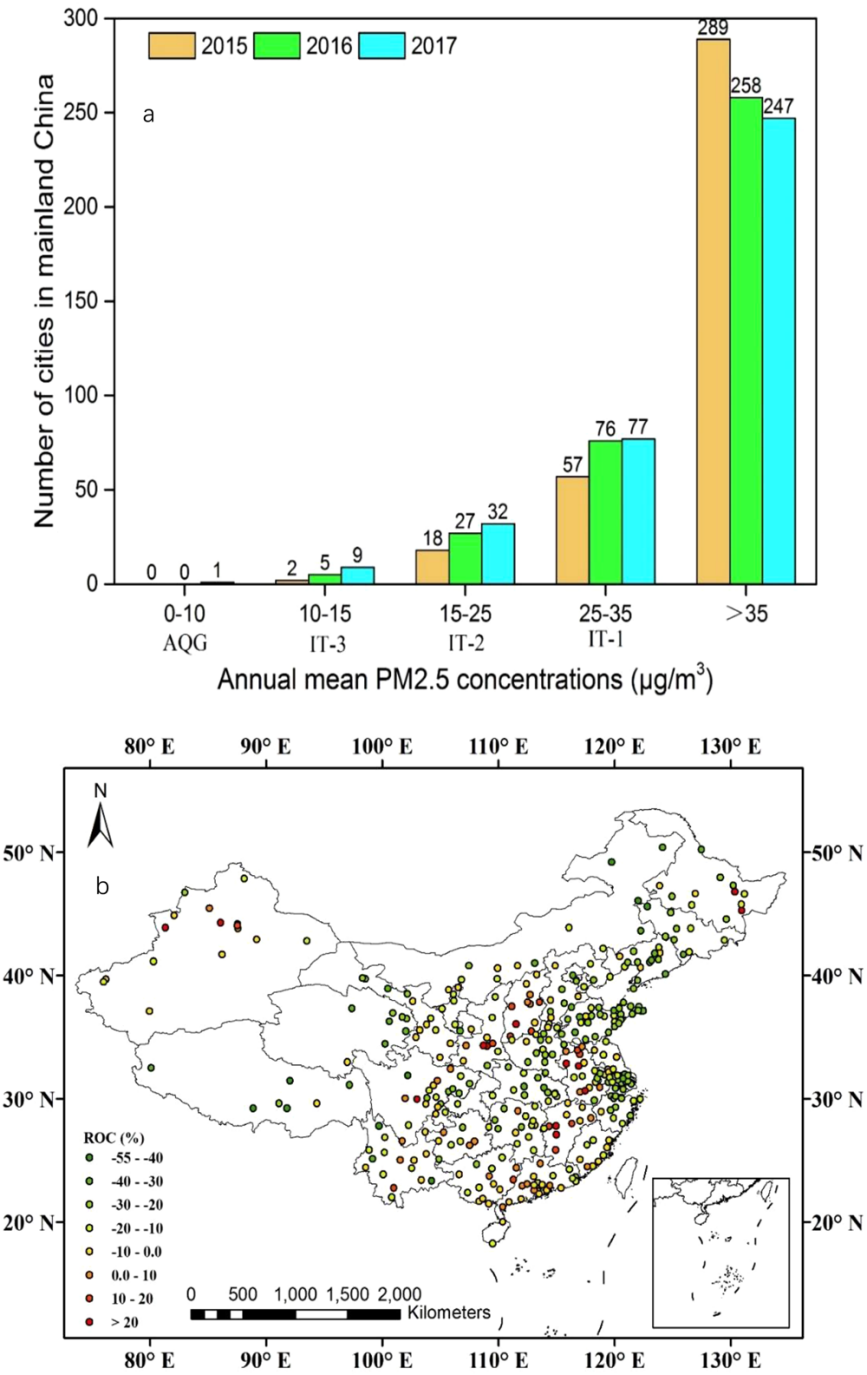
(**a**) Number of cities with annual mean PM2.5 concentrations (μg/m^3^) that met various WHO guideline thresholds during 2015–2017. (**b**) Regional differences in annual mean PM2.5 concentrations (μg/m^3^) over mainland China between 2017 and 2015.

Figure 2a shows the number of cities with annual mean PM2.5 concentrations that met various World Health Organization (WHO) guideline thresholds during 2015–2017. As the figure shows, one can find that the number of cities that met WHO air-quality guidelines (AQG, 0–10 μg/m^3^) increased from zero in 2015 to one in 2017, and those that met WHO interim target-1 (IT-1, 25–35 μg/m^3^) increased from 57 in 2015 to 77 in 2017. Furthermore, compared with 2015, there were 309 cities with annual mean PM2.5 concentrations that had decreased in 2017 (Fig. 2b).

#### Variations of PM10 concentration

**Figure 3:**
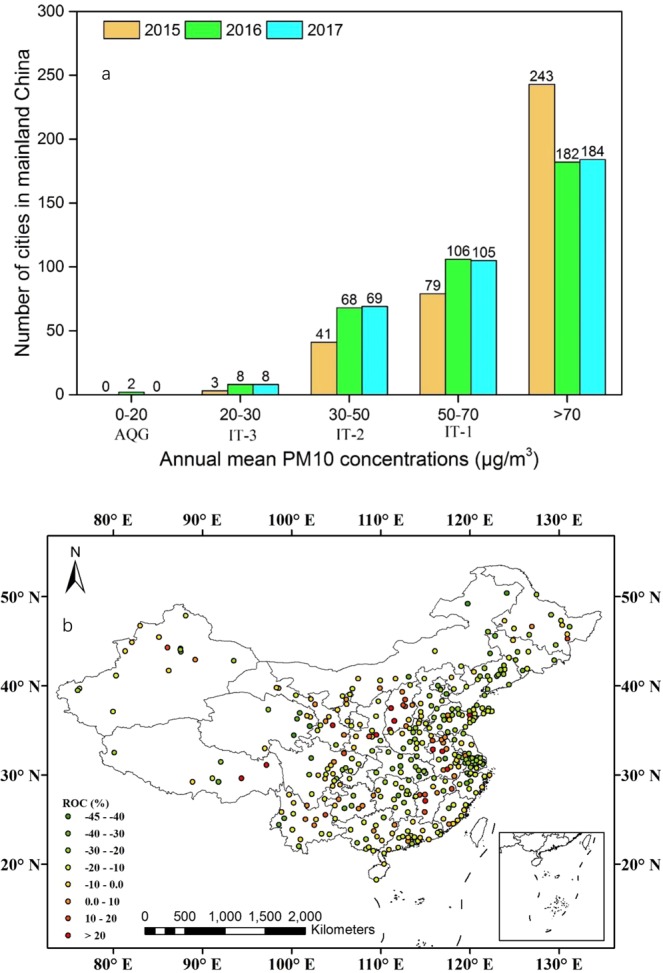
(**a**) Number of cities with annual mean PM10 concentrations (μg/m^3^) that met various WHO guideline thresholds during 2015–2017. (**b**) Regional differences in annual mean PM10 concentrations (μg/m^3^) over mainland China between 2017 and 2015.

As Fig. 3a shows, the number of cities with annual mean PM10 concentrations that met the WHO IT-3 limit of 30 μg/m^3^ increased from three in 2015 to eight in 2017, and that with a value smaller than 70 μg/m^3^ increased from 123 in 2015 to 182 in 2017. Additionally, compared with 2015, there were 309 cities with annual mean PM10 concentrations that had decreased in 2017.

#### Variations of SO_2_ concentration

**Figure 4:**
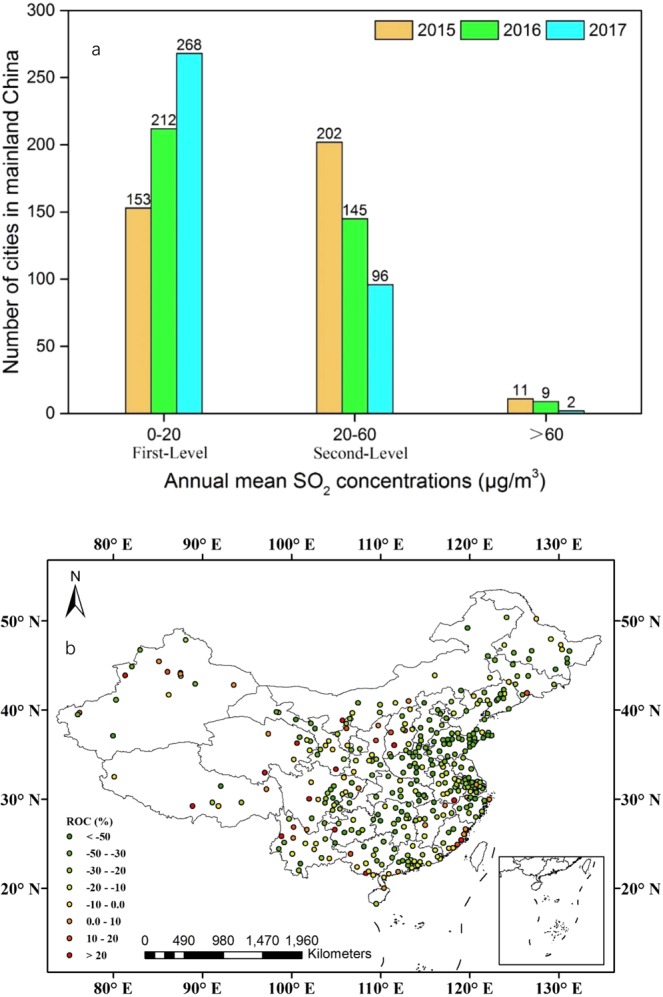
(**a**) Number of cities with annual mean SO_2_ concentrations (μg/m^3^) that met various guideline thresholds of GB 3095–2012 during 2015–2017. (**b**) Regional differences in annual mean SO_2_ concentrations (μg/m^3^) over mainland China between 2017 and 2015.

As Fig. 4a shows, the number of cities that met the first-level concentration limit of 20 μg/m^3^ (GB 3095-2012) increased from 153 in 2015 to 268 in 2017, while that exceeding the second-level concentration limit of 60 μg/m^3^ (GB 3095-2012) decreased from 11 in 2015 to two in 2017. Additionally, compared with 2015, there were 325 cities with a decrease in annual mean SO_2_ concentrations in 2017 (Fig. 4b).

#### Variations of NO_2_ concentration

**Figure 5:**
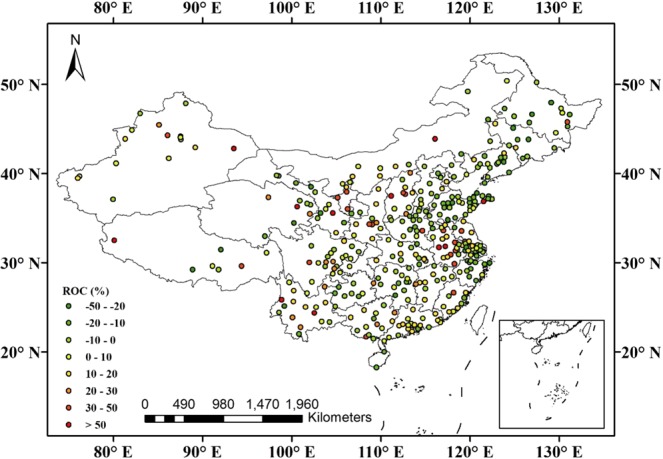
Regional differences in annual mean NO_2_ concentrations (μg/m^3^) over mainland China between 2017 and 2015.

The variations of annual mean NO_2_ concentrations remained relatively stable during 2015–2017, with 2016 (28.8 μg/m^3^) decreasing by 1.6% from 2015 (29.3 μg/m^3^), while increasing by 3.4% in 2017 (29.8 μg/m^3^) compared with 2016 (Fig. 1a). Further analysis indicated that the number of cities that met the first-level concentration limit of 40 μg/m^3^ (GB 3095-2012) increased from 299 in 2015 to 301 in 2017 (Fig. 5).

#### Variations of CO concentration

**Figure 6:**
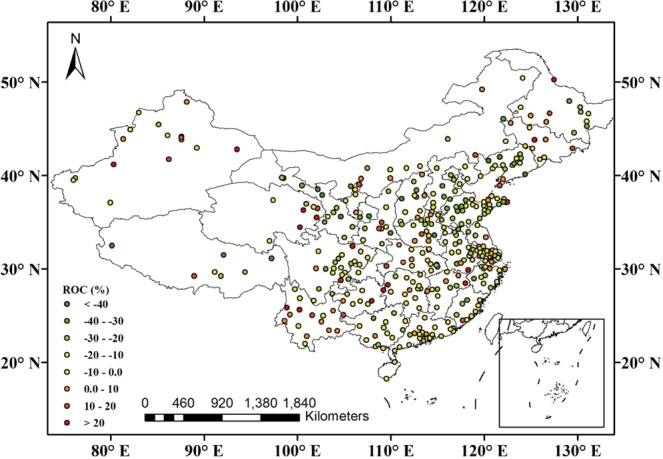
Regional differences in annual mean CO concentration (mg/m^3^) over mainland China between 2017 and 2015.

From Fig. 1a, one can find that the annual mean CO concentration decreased year by year, dropping from 1.10 mg/m^3^ in 2015 to 0.99 mg/m^3^ in 2016 and 0.94 mg/m^3^ in 2017. Figure 6 shows that the number of cities with annual mean mass concentrations of less than 1.0 mg/m^3^ increased from 181 in 2015 to 241 in 2017. Compared with annual mean CO concentration in 2015, there were 285 cities with a value that had decreased in 2017.

#### Variations of O_3_ concentration

**Figure 7:**
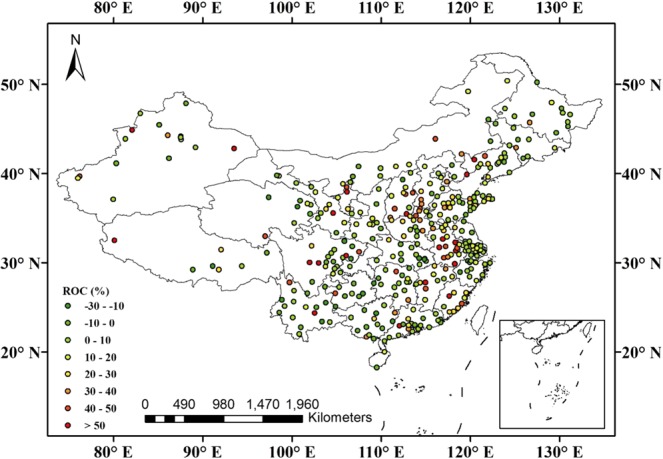
Regional differences in annual mean O_3_ concentration (μg/m^3^) over mainland China between 2017 and 2015.

O_3_ is a secondary pollutant, formed in the atmosphere through the photochemical reactions of NO_*x*_ and volatile organic compounds (VOCs) affected by precursor emissions, solar radiation, and other meteorological factors^18,19^. Figure 7 shows that the number of cities with annual mean O_3_ concentration less than 40.0 μg/m^3^ decreased from 24 in 2015 to 7 in 2017, and those with a concentration greater than 60.0 μg/m^3^ increased from 133 in 2015 to 222 in 2017. In addition, compared with the annual mean O_3_ concentrations in 2015, the number of cities with a decrease in 2017 reached 83, while there were 283 cities with an increase (Fig. 7), suggesting that O_3_ should become the new focus of air-pollution prevention and treatment.

### Natural reasons for variations in air quality

**Figure 8:**
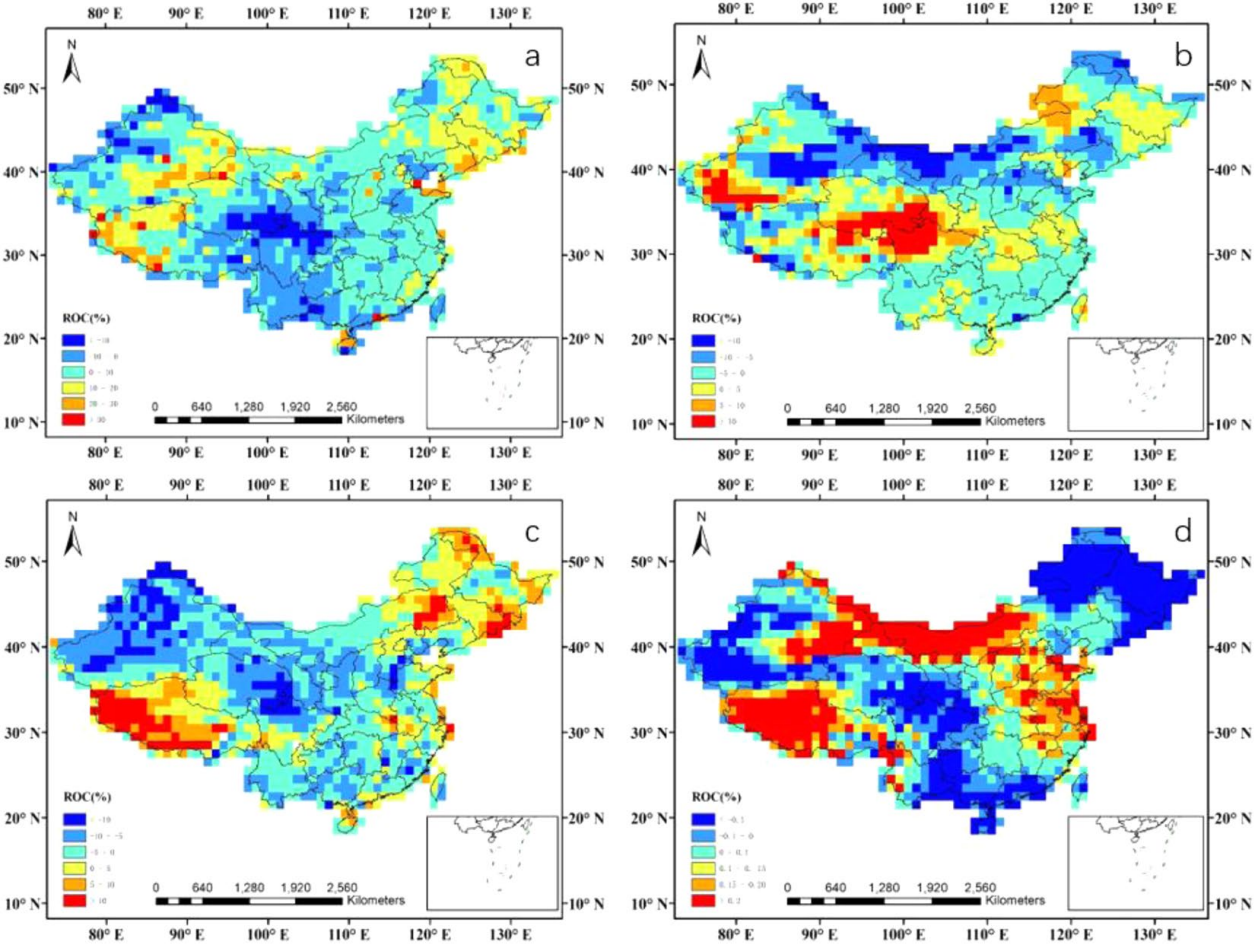
Regional differences over mainland China between 2017 and 2015. (**a**) Planetary boundary layer height. (**b**) Relative humidity. (**c**) Wind speed. (**d**) Air temperature.

The variations in air quality are due to both natural and anthropogenic reasons. The planetary boundary layer (HPBL), relative humidity (RH), wind speed (WS), and air temperature (AT) are the most important meteorological factors influencing air quality. From Fig. 8, one can find that the variations of annual mean value for HPBL were small, while the rate of change (ROC) values were mainly between −10% and 10%, and the annual mean values for HPBL were 494.4, 495.8, and 519.0 m for 2015, 2016, and 2017, respectively. This means that the variations of HPBL may be not have a significant impact on the variations in air quality. The ROC values of RH and WS were also mainly between −10% and 10%, and there were large percentages of areas over mainland China with ROC values between −5% and 5%. In addition, the annual mean values for RH were 57.2%, 57.7%, and 56.5% for 2015, 2016, and 2017, respectively, while that for WS was 4.8 m/s during 2015–2017. Little change in AT was found during 2015–2017, while the ROC values ranged mainly between −0.1% and 0.2% and the annual mean value for AT was 280.5 K during 2015–2017.However, there are differences of HPBL, RH, WS, and AT in different seasons during 2015–2017. More favorable meteorological conditions in winter 2017 than that in 2015, such as higher HPBL values, lower RH values, and faster WS (Figs S1–S5). Thus, for the annual variations of the six air pollutants, based on the above analysis, we can infer that meteorological factors have small influence on the changes of air quality, so what were the main causes of the changes in air quality?

### Anthropogenic reasons for variations in air quality

**Figure 9:**
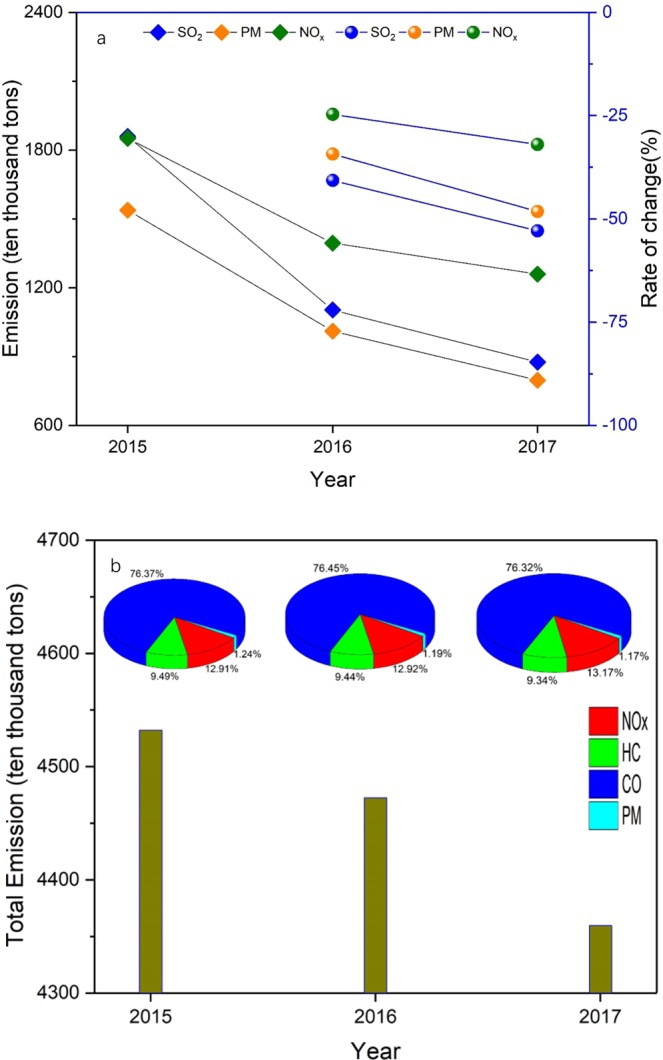
(**a**) Total emissions of air pollutants over mainland China during 2015–2017. (**b**) Total amount of air pollutants discharged by motor vehicles over mainland China during 2015–2017.

The variations in annual mean PM10 concentrations were similar to that of PM2.5 in most cities over China during 2015–2017. The results indicated that PM2.5 accounted for a large fraction of PM10 in most cities (>50%), leading to similar trends between PM2.5 and PM10, in agreement with the findings of Wang *et al*.^7^ and Zhang *et al*.^13^. The maximums of both the annual mean PM2.5 and PM10 concentrations were found in Xinjiang Province, and were determined to primarily be the result of mineral dust from the Taklimakan Desert^20–22^. Interestingly, the increased annual mean PM2.5/PM10 concentrations were mainly in Anhui, Shanxi, Jiangxi, and Guangdong Provinces, consistent with the variations of AQI. It can be inferred that PM2.5 and PM10 may be the major pollutants contributing the most to air quality. To effectively control atmospheric environmental pollution, new stricter environmental protection laws were put into place and enforced in China since 1 January 2015 ^23,24^. The Statistical Communique of the People’s Republic of China (SCPRC) indicated that the total emissions of particulate matter decreased year by year, from 1538.0 ten thousand tons in 2015 to 796.3 ten thousand tons in 2017^25–27^. Compared with 2015, it fell by 48.2% in 2017, which was the main reason for the downward trend of annual mean PM2.5/PM10 concentration (Fig. 9a).

The sources of SO_2_ pollution are classified as both natural and anthropogenic sources, with the latter estimated to account for more than 70% of SO_2_ global emissions^28^. Statistics indicated that total emissions of SO_2_ decreased year by year, from 1859.1 ten thousand tons in 2015 to 1102.9 ten thousand tons in 2016, and then to 875.4 ten thousand tons in 2017. Compared with 2015, SO_2_ emissions fell by 52.9% in 2017 (Fig. 9a)^25–27^. Coal burning was the main anthropogenic source of SO_2_. Therefore, the downward trend of SO_2_ pollution in China was mainly due to government efforts to restrain emissions from coal consumption and the effectiveness of flue-gas desulfurization devices in reducing SO_2_ emissions^29,30^.

Fossil fuel combustion, agricultural production, and motor vehicle emissions are the main sources of NO_*x*_ emissions^31,32^. Emission-reduction measures, especially of reduced vehicle NO_*x*_ emissions, could decrease NO_2_ concentrations significantly^33^. NO_*x*_ emissions accounted for ~13% of total emissions from motor vehicles over mainland China during 2015–2017, with values of 584.9, 577.8, 574.3 ten thousand tons for 2015, 2016, and 2017, respectively (Fig. 9b)^34–36^, which may be related to the variations of annual mean NO_2_ concentration over mainland China.

Motor vehicle emissions are one of the main sources of CO emissions, which account for ~76% of total motor vehicle emissions. Statistics indicated that CO emissions from motor vehicles decreased year by year, from 3461.1 ten thousand tons in 2015 to 3419.3 ten thousand tons in 2016, and then to 3327.3 ten thousand tons in 2017. Compared with 2015, CO emissions fell by 3.9% in 2017 (Fig. 9b)^34–36^, which may mainly account for the downward trend of annual mean CO concentration. O_3_ formation rate mainly depends on the photochemical reactions of NO_*x*_ and VOCs. Although NO_*x*_ concentrations have decreased year by year from 2015 to 2017, O_3_ formation was also affected by the intensity of solar radiation^5,7^.

### Correlations between six air pollutants under different AQI ranges

Based on three years’ daily mean concentrations during 2015–2017, the Pearson’s correlation coefficients (R) were calculated, and the correlations between AQI and six air pollutants under different AQI ranges were obtained (Tables 1 and S2). When the air quality was excellent or pollution severe (Table S1), the correlations between AQI and PM10 were the best (R = 0.85 and 0.48, respectively), followed by PM2.5. Additionally, when the air quality was categorized as good, slight pollution, moderate pollution, heavy pollution (Table S1), the correlations between AQI and PM2.5 were the best (R = 0.81, 0.65, 0.44, and 0.46), followed by PM10. This further suggested that PM2.5 and PM10 were the main factors influencing air quality. The results are different from Yan *et al*.^17^, which indicated that PM2.5 was the major pollutant in China and in Beijing.

**Table 1:** Correlations between six air pollutants under different AQI values.

	0–50						51–100					
	PM2.5	PM10	SO_2_	NO_2_	CO	O_3_	PM2.5	PM10	SO_2_	NO_2_	CO	O_3_
AQI	0.76	0.85	0.21	0.34	0.16	0.30	0.81	0.80	0.23	0.34	0.22	0.02
PM2.5	1	0.59	0.18	0.33	0.24	0.01	1	0.49	0.19	0.32	0.25	−0.15
PM10		1	0.20	0.30	0.11	0.13		1	0.25	0.34	0.17	−0.01
SO_2_			1	0.20	0.18	−0.03			1	0.27	0.26	−0.17
NO_2_				1	0.19	−0.19				1	0.24	−0.32
CO					1	−0.16					1	−0.23
O_3_						1						1
	**101–150**						**151–200**					
AQI	0.65	0.33	0.12	0.15	0.10	−0.12	0.44	0.17	0.06	0.10	0.06	−0.09
PM2.5	1	−0.08	0.14	0.31	0.11	−0.27	1	−0.21	0.16	0.41	0.09	−0.33
PM10		1	0.10	0.06	0.01	0.03		1	0.02	−0.06	−0.04	0.11
SO_2_			1	0.30	0.20	−0.21			1	0.33	0.19	−0.21
NO_2_				1	0.15	−0.36				1	0.15	−0.41
CO					1	−0.16					1	−0.18
O_3_						1						1
	**201–300**						**>300**					
AQI	0.46	0.39	0.05	0.11	0.11	−0.07	0.37	0.48	−0.07	−0.12	−0.03	0.11
PM2.5	1	−0.13	0.21	0.52	0.25	−0.45	1	0.47	0.14	0.36	0.33	−0.32
PM10		1	−0.05	−0.22	−0.11	0.26		1	−0.17	−0.32	−0.25	0.25
SO_2_			1	0.36	0.27	−0.23			1	0.42	0.50	−0.34
NO_2_				1	0.35	−0.54				1	0.81	−0.70
CO					1	−0.32					1	−0.65
O_3_						1						1

Significance at the 0.01 level (P < 0.01).

When the air quality was excellent, good, or severe pollution, the correlations between PM2.5 and PM10 were the best (R = 0.59, 0.49, and 0.47, respectively), suggesting that PM2.5 accounted for a large fraction of PM10^12^. When the air quality was slight, moderate, or heavy pollution, the correlations between PM2.5 and NO_2_ were the best (R = 0.31, 0.41, and 0.52, respectively), which indicated that NO_2_ played an important role in the formation of PM2.5^23,32^. Furthermore, at different AQI ranges, the relationship between O_3_ and SO_2_, NO_2_, CO, PM2.5, and PM10 was mainly negative. Among the five air pollutants, the strongest relationship was between O_3_ and NO_2_ (−0.70 ≤ R ≤ −0.19), indicating that NO_2_ was the most important factor among the five air pollutants in the formation of O_3_^12,18^, allowing us to further infer that NO_2_ played an important role in the formation of PM2.5 and O_3_.

## Discussion

In this paper, we analyzed the spatial and temporal variations of air quality and six air pollutants in 366 cities over mainland China during 2015–2017 for the first time to the best of our knowledge. Since new stricter environmental protection laws were put into place and enforced in China beginning on January 1, 2015, the air quality has been greatly improved based on an AQI decrease of 10.0% in 2017 compared with that in 2015, and the annual mean mass concentrations of PM2.5, PM10, SO_2_, and CO all decreased year by year during 2015–2017. Compared with 2015, they decreased by 14.5%, 13.6%, 30.5%, and 14.5% in 2017, respectively. However, the annual mean NO_2_ concentrations were almost unchanged, and the annual mean O_3_ concentrations increased year by year. These results indicate that it was the anthropogenic reasons that were mainly responsible for the variations in air quality. Further analysis suggested that PM2.5 and PM10 were the main factors influencing air quality, while NO_2_ played an important role in the formation of PM2.5 and O_3_. This paper provides a new view for a comprehensive understanding of the current state of air pollution in China, and the findings will provide a theoretical basis for the formulation of future air-pollution control policy in China.

## Methods

### AQI and six air pollutants

Automated monitoring systems were installed to measure the concentrations of PM2.5, PM10, SO_2_, NO_2_, CO, and O_3_ at each site in China by MEP. Instrument operation, maintenance, data assurance, and quality control were conducted in strict adherence to recent versions of the China Environmental Protection Standards, e.g., HJ 653-2013, HJ 654-2013, GB 3095-2012, HJ 633-2012, and HJ 664-2013^37–39^. MEP has published hourly concentrations of PM2.5, PM10, SO_2_, NO_2_, CO, and O_3_ at 74 cities since January 2013 (http://106.37.208.233:20035/). After 2013, the number of cities that released air-quality data gradually increased, numbering 189 cities in 2014. Since then, 366 cities have regularly released air-quality data since 2015. Since far more data was released in 2015 than in 2014 and 2013, those data can better reflect the atmospheric environment over mainland China, therefore, hourly mean concentrations of PM2.5, PM10, SO_2_, NO_2_, CO, and O_3_ from January 2015 to December 2017 were collected from MEP. We selected the stations with fixed locations and in continuous operation from 2015 to 2017. Finally, a total of ~1500 stations in 366 cities were used to assess the variations of AQI and six air pollutants throughout China.

### Meteorological data

Meteorological data, including HPBL (units of m), RH (units of %), WS (units of m/s), and AT (units of K) were obtained from the U.S. National Centers for Environmental Prediction (NCEP) Final (FNL) Operational Model Global Tropospheric Analyses (https://rda.ucar.edu/datasets/ds083.2/). These NCEP FNL Operational Global Analysis data are on 1° × 1° grids prepared operationally every 6 hours. RH is measured at 2 m above ground and WS and air temperature at a specified pressure difference from ground to 30 hPa.

### Analysis methods

AQI is an index representing the status of air quality devised by MEP, which is calculated based on six air pollutants, namely PM2.5, PM10, SO_2_, NO_2_, CO, and O_3_^40^. AQI ranges from 0 to 500, with larger values indicating worse air quality. Furthermore, AQI is divided into six levels based on different scores, i.e., excellent, good, slight pollution, moderate pollution, heavy pollution, and severe pollution^17^ (see Table S1). The citywide mean concentrations of AQI and six air pollutants were calculated by averaging the concentrations at all sites in each city, which is the same method used by the government to report daily concentrations of air pollutants to the public^17^. Finally, the daily and annual mean mass concentrations of AQI and six air pollutants, and the annual mean meteorological factors at national scale, were calculated on the basis of the arithmetic average method. Additionally, we used the Pearson correlation coefficient (IBM SPSS 21) to test the correlations between AQI and the six air pollutants, each dataset comprising simultaneous daily mean concentrations of PM2.5, PM10, SO_2_, NO_2_, CO, and O_3_ during 2015–2017. Furthermore, ROC was used to compare the variance in annual mean values of AQI, PM2.5, PM10, SO_2_, NO_2_, CO, O_3_, HPBL, RH, WS, and AT between 2015 and 2017 throughout China. ROC is defined as:

1 $${\rm{ROC}}=(x-y)/y\times 100 \% $$

where *x* and *y* represent the annual mean values of AQI, PM2.5, PM10, SO_2_, NO_2_, CO, O_3_, HPBL, RH, WS, and AT in 2017 and 2015, respectively.

## Supplementary information

Supplementary Material
