# Supplementary material for: Spatial and temporal variations of air quality and six air pollutants in China during 2015–2017
Source: Sci Rep. 2019 Oct 23;9:15201. doi: 10.1038/s41598-019-50655-6 (PMC6811589; doi:10.1038/s41598-019-50655-6)
Supplement: Supplementary file 1 — Supplementary Material [file 41598_2019_50655_MOESM1_ESM.docx]

**Supplementary Material**

**Spatial and temporal variations of air quality and six air pollutants in China during 2015 - 2017**

Hong Guo^1^, Xingfa Gu^1^, Guoxia Ma^3^, Shuaiyi Shi^1,2^, Wannan Wang^1,2^, XinZuo^1,2^, Xiaochuan Zhang^1,2^

1 State Key Laboratory of Remote Sensing Science, Institute of Remote Sensing and Digital Earth, Chinese Academy of Sciences, Beijing, China

2 University of Chinese Academy of Sciences, Beijing, China

3 Chinese Academy for Environmental Planning, Beijing, China

* Corresponding author, E-mail: [magx@caep.org.cn](mailto:magx@caep.org.cn).

1. Table S1 The range of AQI and the corresponding air quality levels

Table S1 The range of AQI and the corresponding air quality levels

| AQI | Air quality level | Air quality description | Health implications |
| --- | --- | --- | --- |
| 0–50 | I | Excellent | Air quality is considered satisfactory, and air pollution poses little or no risk. |
| 51–100 | II | Good | Air quality is acceptable; however, for some pollutants, there may be a moderate health concern for a very small number of people who are unusually sensitive to air pollution. |
| 101–150 | III | Slight pollution | Members of sensitive group may experience health effects. The general public is not likely to be affected. |
| 151–200 | IV | Moderate pollution | Slight irritations may occur, individuals with breathing or heart problems should reduce outdoor exercise. |
| 201–300 | V | Heavy pollution | Healthy people will be noticeably affected. People with breathing or heart problems will experience reduced endurance in activities. These individuals and elders should remain indoors and restrict activities. |
| >300 | VI | Severe pollution | Healthy people will experience reduced endurance in activities. There may be strong irritations and symptoms and may trigger other illnesses. Elders and the sick should remain indoors and avoid exercise. Healthy individuals should avoid outdoor activities. |

1. Table S2 The number of records for daily mean values of AQI

| Daily mean AQI range | 0–50 | 51–100 | 101–150 | 151–200 | 201–300 | >300 |
| --- | --- | --- | --- | --- | --- | --- |
| Number of records | 137262 | 186238 | 47735 | 14298 | 7433 | 1996 |

1. Fig. S1 Seasonal variations of average values over mainland China between 2017 and 2015


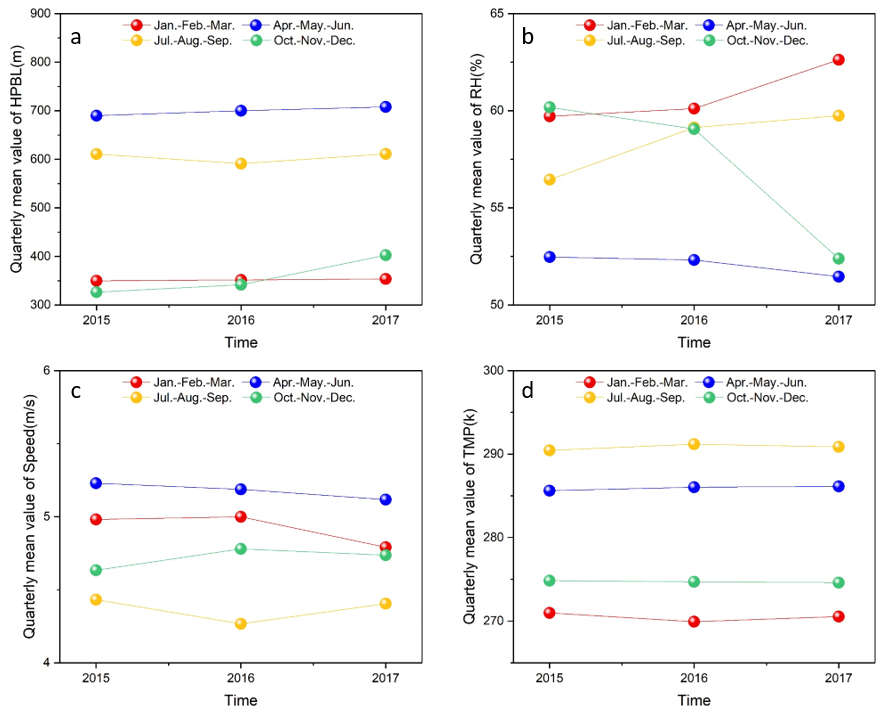


Fig.S1 Seasonal variations of average values over mainland China between 2017 and 2015. a) Planetary boundary layer height. b) Relative humidity. c) Wind speed. d) Air temperature.

1. Fig. S2 Regional differences for seasonal variations of HPBL over mainland China between 2017 and 2015


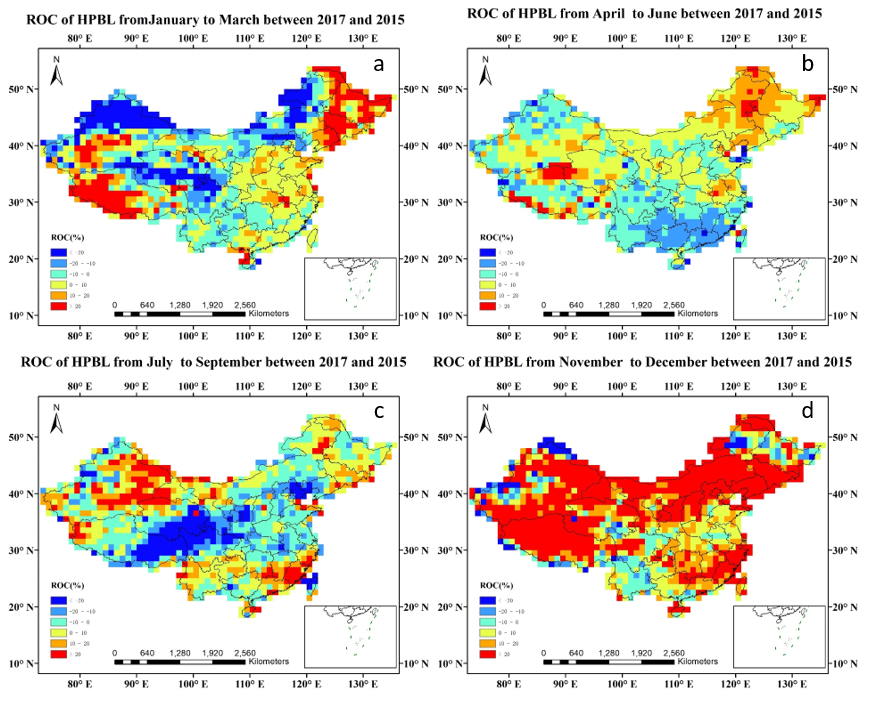


Fig. S2. Regional differences for seasonal variations of HPBL over mainland China between 2017 and 2015. a) Jan.-Feb.-Mar. b) Apr.-May-Jun. c) Jul.-Aug.-Sep. d) Oct.-Nov.-Dec.

1. Fig. S3 Regional differences for seasonal variations of RH over mainland China between 2017 and 2015


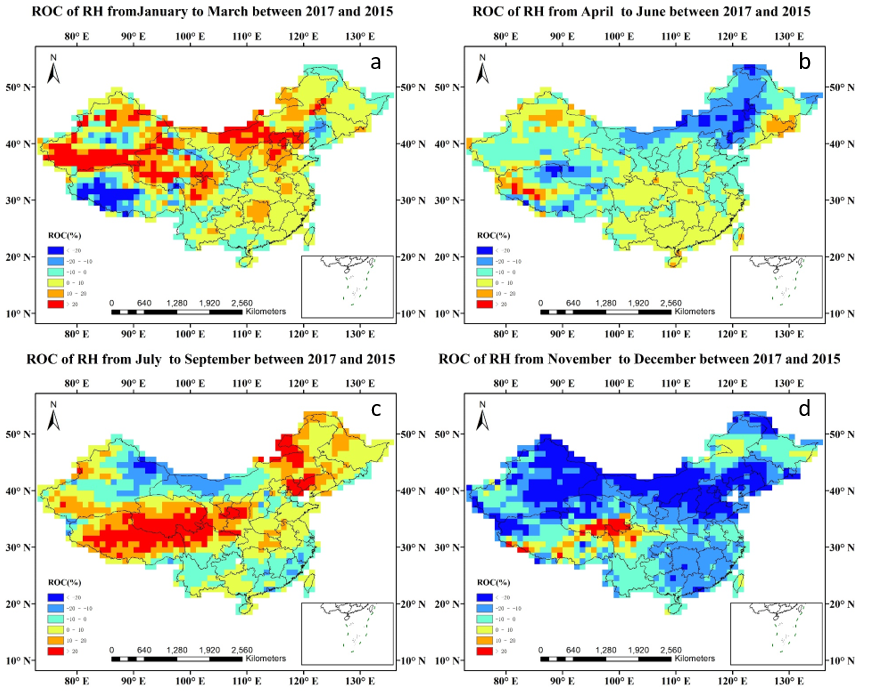


Fig. S3 Regional differences for seasonal variations of RH over mainland China between 2017 and 2015. a) Jan.-Feb.-Mar. b) Apr.-May-Jun. c) Jul.-Aug.-Sep. d) Oct.-Nov.-Dec.

1. Fig. S4 Regional differences for seasonal variations of WS over mainland China between 2017 and 2015


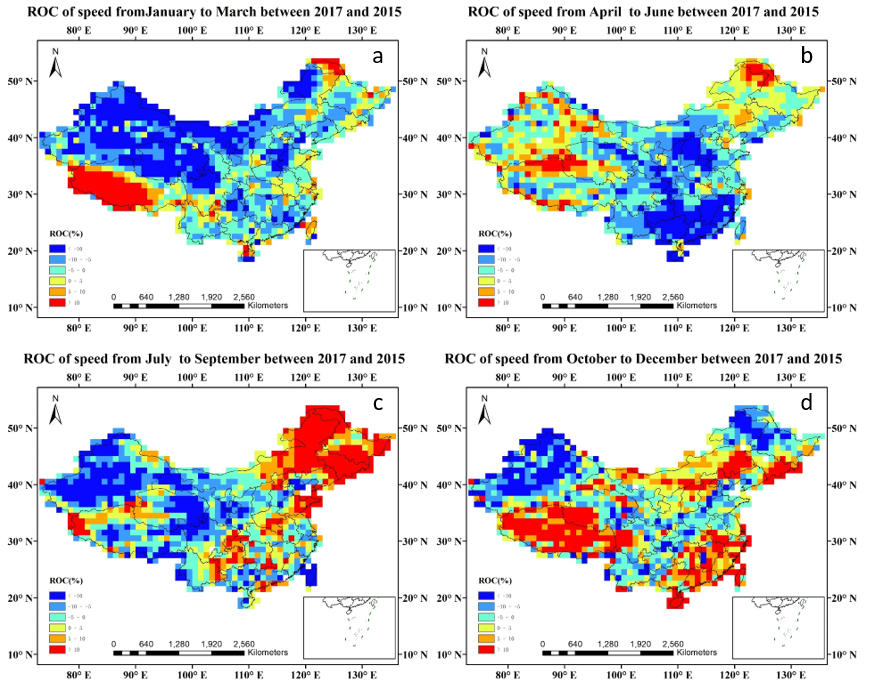


Fig. S4 Regional differences for seasonal variations of WS over mainland China between 2017 and 2015. a) Jan.-Feb.-Mar. b) Apr.-May-Jun. c) Jul.-Aug.-Sep. d) Oct.-Nov.-Dec.

1. Fig. S5 Regional differences for seasonal variations of AT over mainland China between 2017 and 2015


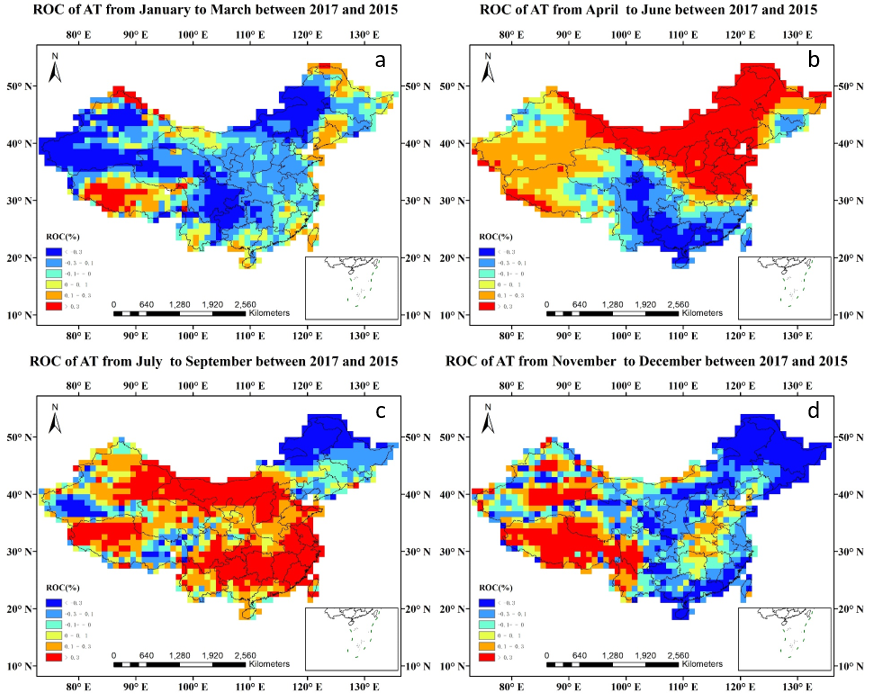


Fig. S5 Regional differences for seasonal variations of AT over mainland China between 2017 and 2015. a) Jan.-Feb.-Mar. b) Apr.-May-Jun. c) Jul.-Aug.-Sep. d) Oct.-Nov.-Dec.
